# Supplementary material for: An In-Silico Comparative Study of Lipases from the Antarctic Psychrophilic Ciliate Euplotes focardii and the Mesophilic Congeneric Species Euplotes crassus: Insight into Molecular Cold-Adaptation
Source: Mar Drugs. 2021 Jan 27;19(2):67. doi: 10.3390/md19020067 (PMC7912073; doi:10.3390/md19020067)
Supplement: Supplementary file 1 [file marinedrugs-19-00067-s001.pdf]

Table S1 Summary of the three lipase families isolated from *E. focardii* and *E. crassus*

	E. focardii		*E. crassus*		Overall sequence identity
	Numbers of proteins	Mean sequence length	Numbers of proteins	Mean sequence length	
Patatin-like phospholipases	9	358.4	17	355.4	0.74
αβ-hydrolase associated lipases	29	392.3	28	386.7	0.69
Esterase lipases	8	294.5	13	286.8	0.53
Total selected lipases	46	-	58	-	-
Total analyzed amino acids	16,958	-	20,598	-	-

Table S2. **Active sites and conserved motifs of lipases from *E. focardii* and *E. crassus*.** A: patatin-like phospholipases; B: α-hydrolase associated lipases; and C: esterase lipases. Patatin-like phospholipases consist of an active-site dyad instead of the more common Ser-His-Asp (or Glu) triad of other lipolytic enzymes showed in B and C. Conserved motives and active sites residues were identified by mapping the amino acid sequences into the 3D structure.

A.

***E. focardii* patatin-like phospholipase**	**Active site S**	**Oxyanion hole**	**Active site D**	**AXXXXP motif**
contig16939	GVSAG	CRALTMSGAGALGAYQ	DGG	ASSSIP
contig09019	GVSAG	CRALALSGGGDKGAYE	DGG	ASSSIP
contig16932	GVSIG	CRALVLEGGGDKGAYQ	DGG	ASSAFP
contig17597	GVSIG	CRALVLEGGGDKGAYQ	DGG	ASSAFP
contig15857	GVSVG	CAALVLEAGGDKGAYQ	DGG	ASSSMP
contig17516	GISVG	CRVLSLEGGGDKGAYE	DGG	ASTSMP
contig17158	GVSIG	CYALALQGGGDKASYQ	DGG	ASSAYP
contig17172	GISIG	CYALALEGGGDKGAYQ	DGG	ASSSYP
contig17423	GVSVG	CRILSFSSGTEKVAYQ	DGS	AATALN
***E. crassus* patatin-like phospholipase**				
contig36455	GVSAG	CRALALSGGGDKGAYE	DGG	ASSSIP
contig15766	GVSAG	CRALALSGGGDKGAYE	DGG	ASSSIP
contig25343	GVSAG	CRALALSGGGDKGAYE	DGG	ASSSIP
contig39840	GVSAG	CRALTMSGAGALGAYQ	DGG	ASSSIP
contig48007	GASAG	CRALALSGGGDKGSYQ	DGG	ASSSIP
contig22176	GVSAG	CRALVMSGGGDKGSYE	DGG	ASTAMP
contig58103	GASAG	CRALVMSGGGDKGSYE	DGG	GSSAIP
contig44802	GVSIG	CKALVLEAGGDRGAYQ	DGG	GSSAIP
contig33219	GVSVG	CKALVLEAGGDKGAYQ	DGG	ASSAIP
contig22497	GISVG	CRALVLEGGGDRGAYQ	DGG	GSAAIP
contig08824	GVSVG	CRVLSMEGGGDKGSYE	DGG	ASASMP
contig61296	GVSVG	CRVLSMEGGGDKGSYE	DGG	ASSSMP
contig29412	GVSFG	CRILSLSSGTEKVAYQ	DGS	AATALN
contig34062	GVSFG	CRILSLSSGTEKAAYQ	DGS	AATALN

contig36398	GVGIG	CYALALQGGGDKASYQ	DGG	ASTAYP
contig42480	GVGIG	CYALALQGGGDKASYQ	DGG	ASTAYP
contig06307	GVSVG	CYALALQGGGDKGAYQ	DGG	ASSSFP

B.

***E. focardii* αβ-hydrolase**	**Active site S**	**Active site D**	**active site H**
contig23876	GHSQG	DAL	HADV
contig23870	GHSQG	DAL	HADV
contig15083	GHSQG	DAL	HADM
contig13985	GHSQG	DAL	HCDL
contig10975	GHSQG	DML	HLDY
contig65380	GHSQG	DML	HLDY
contig44580	GHSQG	DML	HLDY
contig13785	GHSQG	DAL	HLDY
contig13662	GHSQG	DVF	HLDY
contig14778	AHSQG	DLL	HMSF
contig15059	AHSQG	DLL	HMSF
contig15471	AHSQG	DLL	HTSF
contig15033	GHSIG	DHI	HYSF
contig14776	GHSMG	DHI	HFSF
contig14630	AHSMG	DRF	HTSF
contig15152	GHSEG	DLL	CASF
contig15183	AHSEG	DLL	HITF
contig15405	GHSQG	DQM	RDAY
contig18090	GHSQG	DEI	RDSF
contig17643	GHSQG	DGL	RDGF
contig13661	GHSQG	DGY	HYSF
contig15501	GYSFG	DLI	HLSF
contig16046	GYSFG	DVV	HFSF
contig17154	GFSFG	DEL	RDSF
contig16094	SNSFG	DNI	HLSF
contig14599	AYSAG	DDI	HQAF
contig21613	IVSHR	DLY	TYGF
contig14913	GHSQG	DLL	HITF
***E. crassus* αβ-hydrolase**			
contig25340	AFSMG	NLI	HYSF
contig43713	AFSMG	DLI	HYGF
contig01119	AFSMG	DLL	HTGF
contig00786	SYSFG	DIL	HSGL
contig41623	AYSLG	NEI	HFGF
contig00476	GYSFG	DLL	HFGF
contig40952	GYSSG	DQI	HFSF
contig01197	GFSSG	DQI	HFSF
contig15415	GYSSG	DQM	LLSF

contig09869	GFSSG	DQV	HYSF
contig32393	AMSFG	EKM	HVYE
contig25786	AMSFG	EKL	HVYE
contig09161	AMSFG	ERM	QVYE
contig29209	GHSLG	ERL	HVYE
contig46117	GHSLG	ERL	HVYE
contig27445	AISFG	ERL	HVYE
contig02604	AESFG	DQL	RASW
contig33860	GHSFG	DQL	RASW
contig16619	GHSQG	DIF	HLDY
contig61157	GHSQG	DIF	HLDY
contig02220	GHSQG	DIF	HLDY
contig30778	GHSQG	DIF	HLDY
contig20544	GHSQG	SVL	HGDL
contig03399	AHSQG	EQL	HMSF
contig05430	AHSQG	EQL	HMSF
contig71193	GHSIG	SQL	HYSF
contig35775	AHSQG	SQL	HYSF

C.

***E. focardii* esterase**	**Active site S**	**Active site D**	**active site H**
contig12973	GHSLG	DIVP	HIFY
contig19458	GHSLG	DLVA	HKFY
contig19309	GHSLG	DIVP	HLTY
contig12741	GHSLG	DMVP	HLTY
contig15984	GHSLG	DMIP	HLTY
contig12398	GHSLG	DTIP	HLVY
contig11607	GHSLG	DPVP	HLTY
contig11892	GHSLG	DPVA	HLNY
***E. crassus* esterase**			
contig41423	GHSLG	DVVA	HVAY
contig08405	GHSLG	DIVA	HVEY
contig08545	GHSLG	DLVA	HLKY
contig08240	GHSLG	DIVA	HLNY
contig12539	GHSLG	DLVA	HKRY
contig24397	GHSLG	DIVA	HKFY
contig24804	GHSLG	DIVP	HLTY
contig47990	GHSLG	DIVP	HLTY
contig29354	GHSLG	DIVP	HLTY
contig43659	GHSLG	DIVP	HLTY
contig58153	GHSLG	DIVP	HLTY
contig44057	GHSLG	DIVP	HLTY
contig29209	GHSLG	DIVP	HLTY

Table S3: Amino acid composition (pecentage) in *E. focardii* and *E. crassus* lipases

	**Patatin-like**		**αβ-hydrolase**		**Esterase**	
	E. focardii	*E. crassus*	*E. focardii*	*E. crassus*	*E. focardii*	*E. crassus*
Amino acid	*9 Proteins,* ***3226 residues***	*17 Proteins,* ***6041 residues***	*29 Proteins,* ***11715 residues***	*28 Proteins,* ***10828 residues***	*8 Proteins,* ***2356 residues***	*13 Proteins,* ***3729 residues***
A	7.6	7.3	5.8	5.6	4.88	5.63
C	1.14	1.24	1.03	0.89	1.91	2.06
D	6.97	7.37	6.3	7.08	6.37	6.81
E	6.4	6.55	6.4	6.22	6.83	6.62
F	6.16	4.9	6.22	5.83	6.25	6.27
G	8.05	7.65	6.25	6.7	6.58	6.68
H	1.55	1.44	2.68	2.3	4.5	3.62
I	7.2	6.97	7.23	6.7	7.3	6.54
K	6.6	6.55	4.83	3.62	6.4	6.76
L	8	9	8.86	10.01	7.77	7.80
M	2.38	2.58	2.93	3.1	2.08	2.04
N	5.3	5.16	5.50	5.2	6.03	5.26
P	3.22	3.62	4.44	4.87	3.9	4.3
Q	2.79	2.71	3.35	3.76	3.15	3.03
R	3.53	3.67	3.35	3.77	2.29	2.57
S	6.3	6.6	6.48	6.57	7.09	6.83
T	3.99	4.27	5.6	5.3	5.39	4.32
V	7.25	6.87	5.56	5.22	5.94	6.33
W	1.27	1.46	1.7	1.4	0.13	0.48
Y	4.25	4.07	5.48	5.65	5.22	6
Aliphatic I,L,V	22.44	22.84	21.66	21.95	21	20.67
Aromatics F,W,Y	11.69	10.43	13.35	12.88	11.59	12.79
Positive K,R,H	11.69	11.67	10.88	9.77	13.2	12.95
Negative D,E	13.39	13.92	12.7	13.3	13.2	13.43
Tiny G,A,S	21.94	21.53	18.54	18.9	18.55	19.15

Table S4: non-covalent interactions prediction inside the lipase proteins using the RING 2.0 web server (40). Ef pat: *E. focardii* patatin like lipase; Ec pat: *E. crassus* patatin like lipase; Ef αβ: *E. focardii* αβ-hydrolase; Ec αβ: *E. crassus* αβ-hydrolase; Ef est: *E. focardii* esterase; Ec est: *E. crassus* esterase.

interactions		**Ef pat**	**Ec pat**		**Ef αβ**	**Ec αβ**		**Ef est**	**Ec est**
π-π		**8**	7		**20**	22		**21**	26
π-ionic		**2**	0		**0**	0		**0**	1
ionic-ionic		**20**	24		**13**	9		**5**	4
H-bond		**287**	263		**299**	309		**181**	193
VdW		**315**	341		**453**	461		**285**	338
tot		**632**	635		**785**	801		**492**	562

Table S5: Codon frequency (per thousand) in *E. focardii* and *E. crassus* lipases

Frequencies have been estimated from three representative sequences of each species using http://genomes.urv.es/CAIcal/

	**Patatin-like**		**αβ-hydrolase**		**Esterase**	
	E. focardii	*E. crassus*	*E. focardii*	*E. crassus*	*E. focardii*	*E. crassus*
**K**						
AAA	35.415	40.367	33.926	29.344	35.510	34.146
AAG	19.571	26.606	16.155	38.610	22.910	35.366
**N**						
AAC	17.707	18.349	13.732	17.761	13.746	23.171
AAT	41.007	25.688	48.465	20.849	34.364	30.488
**T**						
ACA	13.048	11.927	16.963	13.900	21.764	10.976
ACG	0.932	0.917	0.808	2.317	2.291	6.098
ACC	5.592	8.257	11.309	10.039	4.582	7.317
ACT	18.639	12.844	25.040	26.255	27.491	29.268
**R**						
AGA	28.891	30.275	19.386	24.710	14.891	18.293
AGG	3.728	5.505	4.847	4.633	3.436	2.439
CGA	0	0	1.616	0.772	1.145	1.220
CGC	0.92	0.917	0	0	0	0
CGG	0	0	0	0.772	0	1.220
CGT	11.864	0	2.423	1.544	2.291	1.220
**S**						
AGC	12.116	8.257	5.654	10.039	10.309	8.537
AGT	14.911	6.422	13.732	21.622	14.891	3.659

TCA	11.184	23.853	13.732	7.722	17.182	7.317
TCC	7.456	9.174	4.039	4.633	6.873	7.317
TCG	0	0.917	2.423	3.861	1.145	0
TCT	20.503	17.431	14.540	4.672	24.055	18.293
**I**						
ATA	13.048	3.670	21.002	11.583	10.309	15.854
ATC	19.571	26.606	17.771	23.938	33.219	18.293
ATT	41.938	28.440	37.964	30.888	32.073	23.171
**Q**						
CAA	27.027	18.349	25.040	18.533	19.473	12.195
CAG	0.932	9.174	6.462	13.900	6.873	10.976
**H**						
CAC	3.728	2.752	2.423	10.039	11.455	4.878
CAT	11.184	16.514	21.809	20.077	29.782	21.951
**P**						
CCA	23.299	19.266	19.386	13.900	16.037	17.073
CCC	0.932	1.835	2.423	0.772	2.291	4.878
CCG	0.932	2.752	0	1.544	0	1.220
CCT	12.116	12.844	24.233	23.938	24.055	18.293
**L**						
CTA	13.048	11.927	9.693	12.355	6.873	17.073
CTC	6.524	11.927	8.885	10.039	8.018	6.098
CTG	0.932	10.092	1.616	3.089	3.436	2.439
CTT	27.027	29.358	21.002	29.344	27.491	25.610
TTA	26.095	17.431	29.887	21.622	27.491	15.854
TTG	8.388	15.596	16.963	14.672	3.436	24.390
**E**						
GAA	45.666	46.789	42.811	39.382	43.528	36.585
GAG	14.911	17.431	20.194	28.571	19.473	23.171
**D**						
GAC	18.639	20.183	12.924	24.710	20.619	15.854
GAT	56.85	59.633	50.889	42.471	48.110	56.098
**A**						
GCA	17.707	15.596	16.963	29.344	4.582	31.707
GCC	13.979	17.431	6.462	10.039	10.309	2.439
GCG	2.796	0.917	2.423	1.544	0	3.659
GCT	41.007	38.532	37.157	33.205	35.510	4.146
**G**						
GGA	41.938	52.294	35.541	33.205	44.674	24.390
GGC	6.524	7.339	8.885	11.583	4.582	9.756
GGG	1.864	6.422	5.654	7.722	4.582	9.756
GGT	26.095	12.844	10.501	11.583	17.182	24.390
**V**						
GTA	18.639	24.771	16.963	6.178	12.600	15.854
GTC	14.911	17.431	12.116	14.672	14.891	12.195
GTG	2.796	7.339	4.847	3.089	6.873	18.293
GTT	40.075	26.606	19.386	20.077	28.637	25.610
**Y**						
TAC	11.184	13.761	15.347	13.127	14.891	20.732
TAT	27.959	23.853	40.388	30.116	40.092	43.902
**C**						
TGA	0.932	0	0	0	0	0
TGC	5.592	9.174	0.808	3.089	4.582	7.317
TGT	5.592	1.835	5.654	6.178	11.455	8.537
**F**						

TTC<br>TTT	23.299<br>33.551	27.523<br>11.927	26.656<br>38.772	33.205<br>30.116	32.073<br>30.928	23.171<br>31.707
**M**<br>ATG	21.435	22.94	33.926	27.027	21.764	23.171
**W**<br>TGG	14.911	17.43	18.578	24.710	2.291	10.976
